# Construction and practice of a four-dimensional integrated one-stop outpatient service model based on patient journey mapping

**DOI:** 10.3389/fpubh.2026.1801326

**Published:** 2026-05-21

**Authors:** Caijiao Zheng, Lifen Hu, Zhixiong Zheng, Shaogeng Chen, Wenling Wang, Liyun Chen, Jiaxin Fang, Junhao Li, Quanling Xu

**Affiliations:** 1 Outpatient Department of Quanzhou First Hospital, Quanzhou, Fujian, China; 2 Office of the President of Quanzhou First Hospital, Quanzhou, Fujian, China; 3 Human Resources Management Department of Quanzhou First Hospital, Quanzhou, Fujian, China; 4 Financial Department of Quanzhou First Hospital, Quanzhou, Fujian, China

**Keywords:** four-dimensional integration, outpatient process optimization, patient experience, patient healthcare, patient journey mapping, service-dominant logic

## Abstract

**Background:**

To address prominent problems such as repeated trips between departments, vertical movement across floors and repeated inquiries among outpatients, optimize medical treatment processes, and improve service efficiency and patient experience. Taking the East Campus of Quanzhou First Hospital in Fujian Province as the practice setting, this study constructed a four-dimensionally integrated one-stop outpatient service model covering space, function, personnel and system based on patient journey mapping, Patient healthcare management and service-dominant logic theory.

**Methods:**

Centering on patients’ full-process medical experience, patient journey maps were drawn through semi-structured interviews, on-site process observations and satisfaction surveys to identify process breakpoints and service pain points. Spatial reconstruction, functional integration, personnel integration and system connectivity were implemented to establish an integrated medical service system featuring multi-department collaboration and multi-skilled positions. A pre-post self-controlled study was adopted to compare differences in outpatient process efficiency, patient experience and resource allocation before and after the intervention.

**Results:**

The four-dimensionally integrated one-stop service model effectively broke down departmental barriers and significantly shortened patients’ non-medical waiting time, with the average waiting time for comprehensive services reduced from 11.28 min to 7.56 min. Service continuity and convenience were notably improved, as outpatient satisfaction increased from 91.95 to 95.15 points, and the complaint rate dropped from 1.82 to 0.65 per 10,000 visits. Human resource allocation was optimized, with window staff reduced from 15 to 11, achieving staff reduction and efficiency improvement.

**Conclusion:**

The four-dimensionally integrated one-stop service model based on patient journey mapping can accurately match patient needs, significantly enhance outpatient service efficiency and medical experience, and provide replicable and scalable practical solutions for outpatient service reform in public hospitals. This model promotes the transformation of hospitals from function-centered to patient-centered care, effectively improves patient satisfaction and sense of gain, optimizes resource allocation, reduces operational costs and enhances hospital governance performance, which is consistent with the policy orientation of high-quality development of public hospitals in the new era (1).

## 1 Introduction

In recent years, the global healthcare system has been accelerating its transformation toward patient-centered care, with sustained systematic reform of healthcare service structures. The core goal is to resolve service fragmentation, improve operational efficiency and enhance patient medical experience. As the core component of China’s medical service system, tertiary Grade A general hospitals generally face prominent contradictions including complex service processes, unsatisfactory patient experience and unbalanced resource efficiency (1). Refined departmental settings and clear functional division within hospitals have brought advantages in professional depth, but also led to fragmentation in outpatient, inpatient and various supportive service processes. As the front-end gateway of medical services, outpatient departments have long suffered from common problems such as process fragmentation, prominent departmental barriers, repeated patient queuing and inadequate cross-departmental collaboration, which directly increase operational costs, extend ineffective waiting time and reduce overall patient experience. Such a maze-like medical experience not only consumes considerable time and energy of patients (2) and triggers anxiety and dissatisfaction, but also raises internal coordination costs, causes unbalanced allocation of key resources such as human resources and physical space, and restricts overall operational efficiency (3). Although the patient-centered service concept has been widely accepted, international studies show that translating theoretical frameworks into sustainable and implementable integrated service models still faces many practical challenges.

Patient journey mapping provides a systematic tool for visualizing service touchpoints, emotional experiences and process pain points (4); patient healthcare management, centered on eliminating non-value-added activities, can significantly improve process efficiency (5); service-dominant logic and value-based healthcare offer theoretical support for process optimization (6). However, existing studies mostly adopt a single application perspective, lacking multi-dimensional integration and real-world empirical evidence, resulting in obvious research gaps. Although some hospitals have attempted to set up guidance desks or simple convenience service centers, such services are mostly limited to superficial functions such as information consultation without achieving one-stop integration of space, function, personnel and system, and lack systematic verification of model effectiveness and sustainability. This study integrates the above theories to construct and empirically evaluate a four-dimensionally integrated one-stop outpatient service model covering space, function, personnel and system. It aims to solve the dilemma of service fragmentation, provide evidence-based references for outpatient service reform in public hospitals, form a replicable paradigm for the transformation from function-oriented to patient-centered service models, and explore innovative paths for continuously improving outpatient service efficiency, quality and operational sustainability.

Quanzhou First Hospital of Fujian Province (hereinafter referred to as “our hospital”), founded in 1936, is now the Affiliated Quanzhou First Hospital of Fujian Medical University, a tertiary Grade A general hospital with three comprehensive campuses and 2,890 authorized beds. In 2024, the hospital recorded 2.19 million outpatient visits and 127,700 discharges. Characterized by a large annual outpatient volume and high proportions of older adults patients (42%), cross-regional medical insurance patients (28%) and special disease patients (15%), our hospital’s traditional outpatient model placed multiple independent windows including the hospital service center, outpatient charging office, medical technology appointment center and medical insurance service station in different areas on the first and second floors, with scattered businesses and independent systems, leading to distinct service pain points. Therefore, starting from 2025, our hospital has comprehensively sorted out outpatient service processes. Guided by patient journey mapping, patient healthcare management and service-dominant logic theory, the hospital accurately identified touchpoints, behavioral characteristics, psychological demands, emotional change curves, as well as potential service pain points and optimization opportunities at each stage of the patient journey (7). For medical services including outpatient consultation, complaint handling, invoice printing, medical record establishment, registration (including appointment registration), charging, refund, medical insurance review, verification and stamping of medical certificates, information correction, medical record copying and centralized examination appointment, the hospital has implemented a model of one-window handling, multi-skilled positions and unified stamping management, aiming to minimize patient movement and maximize service efficiency to effectively improve patients’ medical experience.

## 2 Methods

### 2.1 Literature search

Domestic and foreign research findings in fields including one-stop outpatient services, patient journey mapping, Patient healthcare management and service-dominant logic were systematically reviewed to provide solid theoretical support and policy basis for this study.

### 2.2 Lean medical management

Lean medical management originates from the lean concept and is a systematic operational transformation paradigm for the high-quality development of tertiary Grade A general hospitals (8). Its core is to be guided by patient value, achieve efficient resource allocation and simultaneous improvement of service quality by identifying value streams, eliminating waste, optimizing processes, and continuous improvement (9). It emphasizes creating maximum medical value with minimal input, focusing on eliminating non-value-added links such as waiting, defects, over-processing, ineffective circulation, and manpower idleness, and reconstructing a smooth, safe, and accessible diagnosis and treatment circulation path. In large tertiary hospitals with large patient flow, complex processes, and refined division of labor, lean medical management effectively solves prominent problems such as departmental barriers, process fragmentation, repeated queuing, and resource waste (10).

### 2.3 Service-dominant logic

The core view of service-dominant logic is that medical services are not simply diagnosis and treatment outputs, but dynamic processes in which doctors, patients, and multiple subjects participate together to create value collaboratively. This theory abandons the traditional supply-side thinking of “service provider-centered,” emphasizing that the patient is an active participant in their own health and medical experience, and the core function of medical institutions is to build a value co-creation platform rather than output services unilaterally (11). In the complex outpatient scenario of tertiary general hospitals, service-dominant logic requires breaking departmental barriers and information barriers, integrating resources with the patient’ s full-process needs as the core, and promoting integrated collaboration of processes, personnel, systems, and space.

### 2.4 Patient journey mapping tool and components

As a core service design tool, patient journey mapping visually presents the service touchpoints, interactions, and experience feelings of the patient throughout the whole process from the emergence of health awareness to recovery from the patient’ s perspective. It can systematically transform the patient’ s subjective experience into a structured framework for managers to conduct objective analysis and intervention (12). Its core components include patient persona, journey stage division, touchpoints, behaviors, thoughts, emotional curves, pain points, and opportunities. Among them, patient persona helps to ensure that the analysis focuses on the real needs and background information of specific patient groups; journey stages divide the patient’ s continuous experience into logical paragraphs with clear goals; behaviors and thoughts record the patient’ s external actions and internal psychological activities respectively; pain points identify specific links that cause the patient to feel frustrated, confused or dissatisfied, while opportunities correspond to potential improvement space. These components interact with each other, making the journey map go beyond simple process description and become a key bridge connecting patient experience perception and hospital service design.

Drawing a patient journey map usually follows the following steps: clarifying research objectives, selecting representative patient samples, collecting relevant data through interviews and observations, visualizing journey stages and touchpoints, analyzing patient emotions and pain points, and finally refining insights and exploring design opportunities. In the field of medical service optimization, the application of this theory has shifted from the improvement of a single link to the systematic reconstruction of the whole process (13). By mapping outpatient, inpatient, or inter-institutional referral journeys, key pain points such as difficult appointment, repeated queuing, opaque information, and poor cross-department coordination can be identified, thereby providing an intuitive basis for process redesign and transforming the abstract “patient-centered” concept into operable and measurable specific improvement points (14).

**Figure 1:**
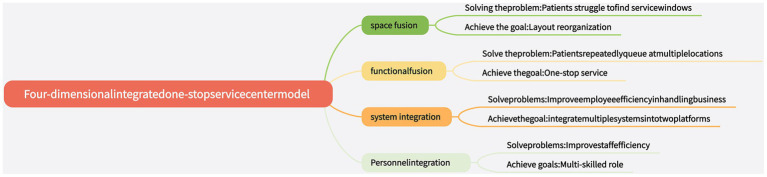
The conceptual framework diagram of the patient journey mapping.

Through analyzing each component in the patient experience stages (S1, S2…, Sn) and presenting them visually, the conceptual framework diagram of the patient journey map was determined (see Figure 1).

### 2.5 Application

This study established a comprehensive theoretical framework by taking patient journey mapping in medical service design as a diagnostic tool, introducing lean management thinking and service-dominant logic. Patients were regarded as the “journey subject” throughout multiple discrete service links rather than passive recipients of services. Meanwhile, the research perspective was shifted from the convenience of internal hospital management to the continuity of external patient experience, providing a systematic visual analytical framework for identifying service pain points in tertiary general hospitals and designing solutions for the one-stop service center.

By mapping the full-journey touchpoints of outpatient patients from the emergence of medical needs to after discharge, the actual movement paths and interactive experiences of patients in key links such as appointment, registration, waiting for consultation, examination, payment, medicine collection, consultation and certificate handling can be clearly traced, directly presenting the breakpoints and emotional lows encountered by patients in non-core medical links. Among them, situations such as patients repeatedly asking for directions due to unfamiliarity with department distribution, queuing at different windows for scattered services, and traveling between multiple departments for stamping to obtain medical certificates will lead to a sharp rise in their confusion, anxiety and dissatisfaction. The touchpoints corresponding to these emotional lows are exactly the “pain points” and “breakpoints” of low efficiency and poor experience caused by fragmented service processes, providing a clear basis for subsequently breaking departmental boundaries and formulating reform measures that meet patient expectations and values (15).

#### 2.5.1 Semi-structured or in-depth interviews

This study adopted the purposive sampling method to gain an in-depth understanding of patients’ experiences, perceptions and needs at different medical stages. A total of 100 patients were selected for semi-structured or in-depth interviews (16). The sample mainly focused on older adults patients, cross-regional medical insurance patients and special disease patients because such groups face the most obstacles and the most complex movement paths in the outpatient process, and are most sensitive to defects in service processes. Analyzing their experiences can maximize the identification of key pain points in the service system and ensure that optimization measures benefit the most vulnerable groups.

#### 2.5.2 Process observation

Researchers personally participated in the medical treatment process of outpatient patients to observe patients’ behaviors and emotional changes and capture their real reactions in the actual environment. The whole medical process of 100 patients (including 50 special disease patients and 50 cross-regional medical insurance patients) was tracked and recorded, and data such as queuing links, handling duration and movement distance were statistically analyzed.

#### 2.5.3 WeChat evaluation survey

A questionnaire based on a 5-point Likert scale was adopted, which presented satisfactory internal consistency. In this sample, the Cronbach’s α coefficient of the total scale was 0.85, and the α coefficient of each dimension was above 0.70, indicating acceptable reliability of the questionnaire (17). The questionnaire covered five dimensions: service attitude, service process, service environment and service quality. A total of 300 valid samples were collected through this questionnaire to understand patients’ satisfaction with outpatient services and collect suggestions for improvement.

**Figure 2:**
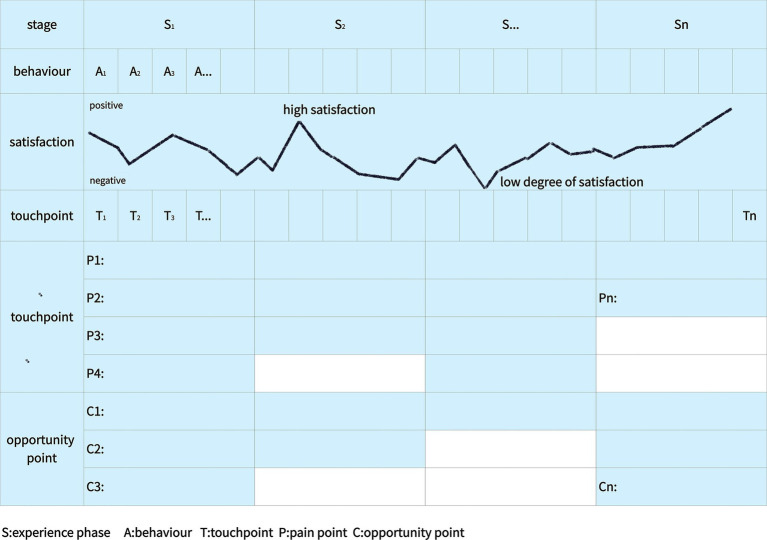
The outpatient patient journey mapping.

The outpatient patient journey map was drawn based on interviews, observations and surveys (see Figure 2).

## 3 Construction and implementation of the four-dimensional integrated one-stop service center model

**Figure 3:**
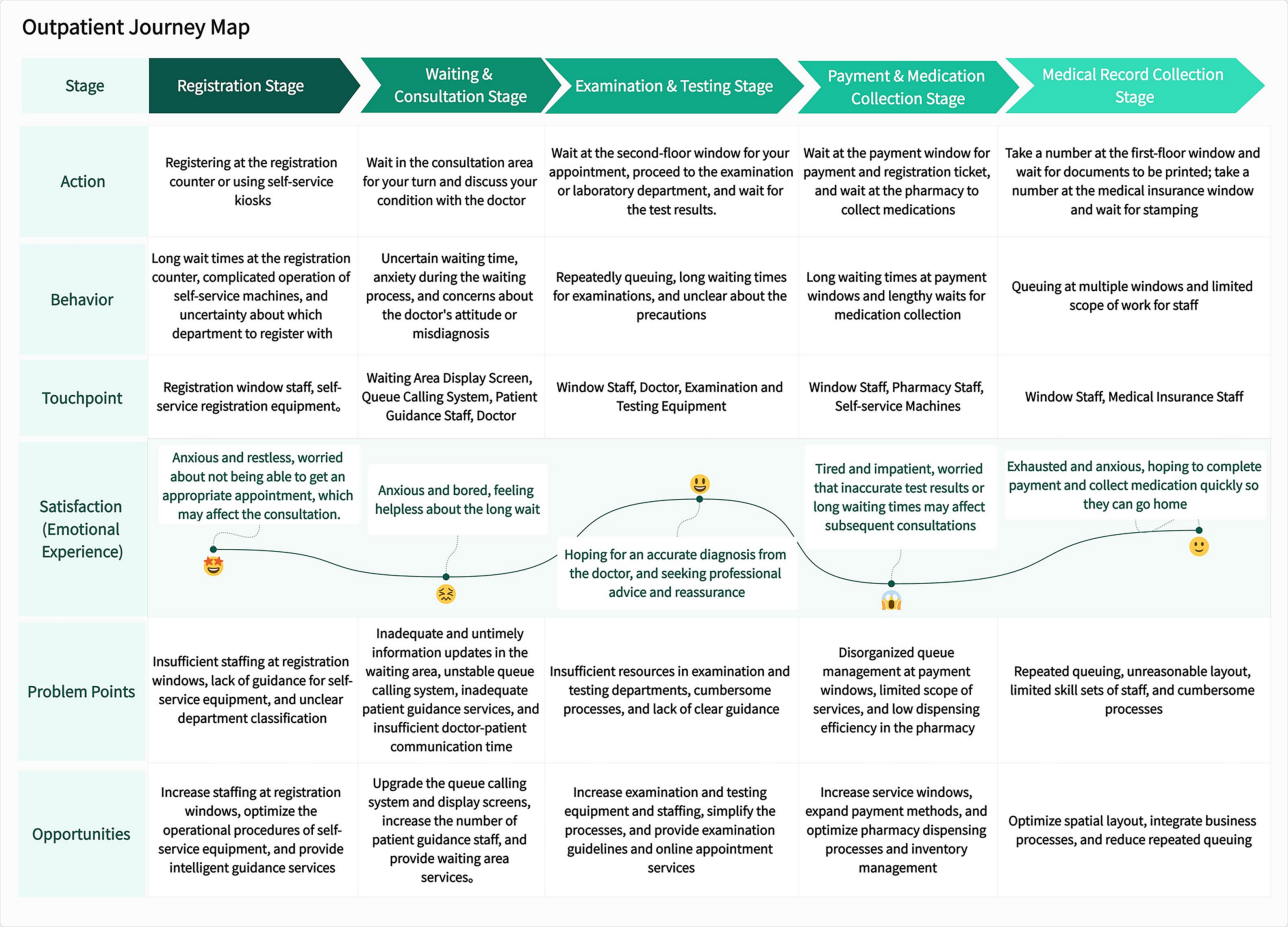
The framework of the four-dimensional integrated one-stop service center.

Guided by the pain points identified through the outpatient patient journey mapping, the framework of the four-dimensional integrated one-stop service center was designed (18) (as shown in Figure 3).

### 3.1 Results of behavioral language model

Through research and sorting out the entire outpatient process of patients from “registration→waiting for consultation→diagnosis→inspection→payment→medicine collection,” a patient journey mapping was drawn to mark behaviors, needs, pain points and emotional changes at each stage, identifying four categories of core pain points as shown in Table 1.

**Table 1:** Identification of core pain points in outpatient services based on patient journey mapping.

Pain point type	Proportion	Core performance
Process pain points	40%	Businesses are not integrated with numerous handling links, and the business processes for special disease patients and cross-regional medical insurance patients are particularly complex.
Spatial pain points	30%	Service windows are scattered, requiring patients to handle businesses across floors with high costs for locating windows and moving around.
Personnel pain points	15%	Staff have single skills and face inter-departmental communication barriers, making it difficult to provide accurate guidance to patients.
System pain points	15%	Systems are independent of each other with no information sharing and cumbersome operation interfaces, resulting in low business handling efficiency.

### 3.2 Design of the four-dimensional integrated one-stop service center model

Guided by the pain points identified through the patient journey mapping, an outpatient one-stop service center model integrating four dimensions space, function, personnel and system was constructed.

#### 3.2.1 Spatial integration

The service layout was reconstructed by centrally integrating the charging office, hospital service center, medical technology appointment center and medical insurance service station, which were previously scattered on the first and second floors, to establish one-stop service centers on both floors. Specifically, the first floor focuses on medical insurance services and comprehensive services, while the second floor focuses on post-consultation settlement and appointment services, thereby minimizing patient movement.

#### 3.2.2 Functional integration

More than 10 businesses including consultation, medical record establishment and registration, charging and refund, medical record copying, inspection appointment, test report printing, medical insurance review and medical certificate stamping were integrated, implementing a model of “one-window acceptance, unified stamping management and multi-functional positions,” with a focus on optimizing the service processes for special disease patients and cross-regional medical insurance patients.

#### 3.2.3 Personnel integration

Eleven staff members were seconded across departments (compared with 15 before integration) and trained through a two-month cross-training program to develop into multi-functional compound talents, mastering non-medical comprehensive skills covering departments such as the Finance Department, Medical Insurance Department, Outpatient Department and Medical Administration Department, with the first-reception responsibility system implemented (19).

#### 3.2.4 Software system integration

The original 10 independent business systems were integrated into 2 software systems: a business system and a centralized printing platform, with data interfaces connected to achieve information sharing (20); in terms of hardware, 10 operation interfaces were integrated into 7, and equipment such as multi-functional card readers were configured to simplify operations.

### 3.3 Implementation steps

The implementation cycle lasted 7 months, advancing in 6 phases as shown in Table 2.

**Table 2:** Six-phase implementation roadmap for the one-stop service model.

Phase	Phase name	Time cycle	Core work content
①	Research and preparation	Month 1	Establish an inter-departmental team and design research tools including in-depth interview outlines, process observation records and patient satisfaction questionnaires.
②	Map drawing	Month 2	Complete in-depth interviews with 100 patients and full-process observation of 300 patients’ medical visits, draw the patient journey mapping and identify core pain points.
③	Scheme design	Month 3	Formulate one-stop service implementation plans including spatial renovation layout scheme, system integration technical scheme and personnel cross-training scheme.
④	Renovation and implementation	Months 4–5	Promote the decoration of one-stop service centers on the first and second floors, complete the integration and commissioning of business systems and hardware equipment, and carry out comprehensive business training for staff.
⑤	Trial operation	Month 6	Pilot operation of the one-stop service center, collect patient feedback and operation data, and dynamically optimize service processes through the PDCA cycle.
⑥	Comprehensive promotion	Month 7	Officially launch the one-stop service in full scale, establish a standardized management system, inter-departmental coordination mechanism and long-term assessment mechanism.

## 4 Results analysis

After the establishment of the one-stop service center, the number of medical treatment links, total duration and window waiting time were all significantly shortened, and patient satisfaction was significantly improved. Paired t-tests showed highly statistically significant differences (all *p* < 0.001). The overall outpatient complaint rate decreased remarkably, and the proportion of complaints related to “cumbersome processes” dropped substantially. Chi-square tests indicated highly statistically significant differences (all *p* < 0.001).

### 4.1 Significant improvement in process efficiency

#### 4.1.1 Queuing links

After the establishment of the one-stop service center, non-diagnostic and treatment process links were significantly reduced. For example, the average number of queuing links for special disease patients decreased from 6 to 3, and for cross-regional medical insurance patients from 5 to 2, both meeting the target of ≤ 3 links, as shown in Table 3.

**Table 3:** Reduction in queuing links for key patient groups before and after optimization.

Patient type	Number of queuing links before optimization	Number of queuing links after optimization	Result
Special disease patients	6	3	≤3
Cross-regional medical insurance patients	5	2	≤3

#### 4.1.2 Total patient consultation duration and window waiting time

The total duration of the entire outpatient consultation process decreased significantly after the establishment of the one-stop service center. For example, the average total processing time for special disease patients was shortened from 66 min to 35 min, representing a reduction of 46.9%; the average total duration for initial visits of cross-regional medical insurance patients was reduced from 79 min to 48 min, a decrease of 39.2%; and the average handling time for comprehensive patient services was shortened by 43.05%. The average waiting time at outpatient charging windows was 11.28 min before the establishment of the one-stop service center, which fell to 7.56 min afterward, a reduction of 32.97%. More details as shown in Table 4.

**Table 4:** Total patient consultation duration and window waiting time.

Indicator	Before optimization	After optimization	Reduction rate	t-value	*p*-value
Total duration of the whole consultation process for all patients	95.00 min	68.00 min	28.40%	12.68	<0.001
Processing duration for special disease patients	66.00 min	35.00 min	46.90%	15.32	<0.001
Duration for initial visits of cross-regional medical insurance patients	79.00 min	48.00 min	39.20%	13.17	<0.001
Average window waiting time	11.28 min	7.56 min	32.97%	9.84	<0.001

### 4.2 Continuous improvement in patient experience

After the establishment of the one-stop service center, the patient complaint rate decreased from 1.82 cases per 10,000 person-times to 0.65 cases per 10,000 person-times, a reduction of 64.28%, among which the proportion of complaints related to “cumbersome processes” dropped from 70 to 10%. In terms of patient satisfaction, the overall outpatient satisfaction increased from 91.95 points to 95.15 points, the score for the “process convenience” dimension rose from 90.02 points to 94.13 points, and the satisfaction of cross-regional medical insurance patients and special disease patients increased from 89.97 points and 89.95 points to 94.80 points and 93.36 points, respectively. More details as shown in Tables 5, 6.

**Table 5:** Reduction in patient complaint rates and complaint categories.

Indicator	Before optimization	After optimization	Reduction range	$X^{2}$ -value	*p*-value
Outpatient complaint rate (cases per 10,000 person-times)	1.82	0.65	64.28%	54.48	7.26
Proportion of “cumbersome process” complaints	70.00%	10.00%	60.00%	68.24	9.45

**Table 6:** Improvement in patient satisfaction scores across key dimensions.

Satisfaction indicator	Score before optimization	Score after optimization	Increase range	t-value	*p*-value
Overall outpatient satisfaction	91.95 points	95.15 points	3.20 points	7.26	<0.001
Satisfaction with “process convenience” dimension	90.02 points	94.13 points	4.11 points	9.45	<0.001
Satisfaction of cross-regional medical insurance patients	89.97 points	94.80 points	4.83 points	11.02	<0.001
Satisfaction of special disease patients	89.85 points	93.36 points	3.51 points	7.83	<0.001

### 4.3 Resource allocation optimization and intangible benefits

Human Resource Optimization: The proposed “four-dimensional integration” model responds to the call for improving resource allocation efficiency by integrating human and spatial resources. It demonstrates that with the same service volume, the number of staff was reduced by 4 persons, and the per capita business volume increased by 36.4%, achieving “staff reduction and efficiency improvement.” Service quality and patient health outcomes can also be significantly improved through management innovation (21).

Other benefits: Staff professional capabilities and inter-departmental collaboration efficiency were significantly improved, a standardized new outpatient service management model was constructed, the hospital’s brand influence was expanded, and it attracted many sister hospitals to visit and learn.

## 5 Conclusion

### 5.1 Resolving fragmented medical processes and enhancing patient experience

The traditional scattered service processes in public hospitals, characterized by “three longs and one short” and repeated trips for patients, represent key bottlenecks targeted in national medical reform. The one-stop service center integrates high-frequency functions such as consultation, appointment and settlement, transforming serial processes into parallel single-point services. It reduces ineffective waiting and physical exertion for patients, alleviates anxiety caused by information asymmetry through full-process guidance by service specialists, implements the “patient-centered” concept, and enhances patients’ sense of gain and satisfaction with medical treatment.

### 5.2 Breaking departmental barriers and optimizing hospital operational efficiency

Resource waste and high coordination costs caused by departmental barriers hinder refined hospital management (22). With standardized processes and centralized resource scheduling, the one-stop service center eliminates non-value-added links such as repeated queuing and duplicate information entry, acting as a hub for cross-departmental collaboration. It drives hospitals to shift from a “department-oriented” model to a “process-oriented” model, improving service efficiency, cutting operational costs, and consolidating the internal governance foundation for high-quality hospital development. At the management level, this model reduces internal coordination costs and releases human resources through resource integration, allowing medical staff to focus on core medical services and enhancing the hospital’s overall operational efficiency and core competitiveness (23).

### 5.3 Responding to medical reform policies and promoting service model transformation

National deepened medical reform explicitly requires a shift from “disease-centered” to “health-centered” care, with improving medical services as a core task. The one-stop service center is not merely a process optimization, but a carrier that translates macro medical reform policies into micro service changes. By reconstructing the patient’s medical journey and resolving medical difficulties, it fulfills the requirements of “improving medical experience and enhancing patient experience,” representing a key practice for public hospitals to follow the direction of medical reform and providing a replicable response path for other hospitals (24).

### 5.4 Strengthening public welfare attributes and elevating medical public value

The core mission of public hospitals is to safeguard social health and well-being, while traditional cumbersome medical processes impair social benefits. The one-stop service center strengthens the public welfare attributes of public hospitals by reducing patients’ non-medical costs and improving service accessibility and friendliness. As a unified service interface and collaborative hub, it enhances the hospital’s ability to respond to complex needs, shapes the image of a responsible public institution, further elevates the public governance level of the medical and health system, and helps maximize the public welfare value of medical services (25).

## 6 Discussion

### 6.1 Consistency with theories

This study confirms that the four-dimensionally integrated one-stop model based on patient journey mapping effectively resolves core pain points including outpatient fragmentation, complex processes and high coordination costs, which is highly consistent with international theories of lean healthcare and service-dominant logic. Patient journey mapping intuitively reveals service breakpoints and accurately identifies non-value-added activities, in line with the waste elimination principle of lean healthcare. One-stop integration conforms to the user-centered core concept of service design. One-window handling and data interoperability embody the value co-creation connotation of service-dominant logic (26). The simultaneous improvement of efficiency and experience accords with the direction of value-based healthcare reform, further enriching the application scenarios of medical service design theory.

### 6.2 Comparison with international literature

The results are consistent with international evidence that integrated services can significantly shorten patient waiting time, improve satisfaction and reduce hospital operational costs. Compared with existing studies, the innovations of this study lie in four aspects: 1. It proposes for the first time a four-dimensional integration framework of space-function-personnel-system, breaking through the limitation of single-dimension process optimization; 2. It adopts patient journey mapping throughout the whole process of design and evaluation, forming a closed loop of pain point identification, scheme design and effect verification; 3. It achieves synergistic improvement in four dimensions: efficiency, experience, cost and human resources, avoiding the imbalance caused by single-indicator optimization; 4. It provides a replicable outpatient reform plan for Chinese tertiary hospitals, suitable for the management scenario of public hospitals and patient needs in China, filling the application gap of international research in China’s medical context.

### 6.3 Sustainability and implementation value of the model

The four-dimensional integration model features long-term sustainability supported by four core factors: first, low one-time renovation investment, with long-term labor cost savings through process optimization; preliminary estimation shows that the initial investment in system renovation and decoration can be recovered within 1–2 years through labor cost reduction. Second, standardized processes and integrated systems reduce hospital management complexity and minimize human errors. Third, the consolidated inter-departmental collaboration mechanism lowers the risk of reform rebound. Fourth, it can be connected to AI, intelligent guidance and data middle platforms for digital upgrading to continuously enhance adaptability. This model exerts positive externalities on health system performance, medical financing efficiency and service accessibility, consistent with global health system improvement pathways, and can support the optimization of regional medical and health systems (27).

### 6.4 Operational risks and limiting factors for promotion

Certain risks exist in the operation of the four-dimensional integration model, mainly concentrated in the management of multi-skilled staff. On the one hand, employees are required to master multi-post skills with increased workload, and long-term high-intensity work may lead to declined service quality and job burnout. On the other hand, turnover of key staff may disrupt business continuity and affect service consistency. For promotion, the model demands a high level of hospital informatization to realize interconnection of multiple systems, making it difficult for hospitals with weak informatization foundations to adapt quickly. Meanwhile, strong inter-departmental coordination capacity of management is required to break departmental barriers, posing greater promotion challenges for hospitals with ambiguous departmental authority and incomplete collaboration mechanisms.

### 6.5 Implications for public health policy

This study carries important implications for public health policy: first, it provides empirical support and technical pathways for implementing the national policy of “improving medical experience and enhancing patient experience,” proving that process integration and service optimization can effectively resolve medical bottlenecks. Second, the four-dimensional integration model empirically demonstrates that process optimization can improve the utilization efficiency of medical resources, offering decision-making evidence for health administrative departments to formulate hierarchical diagnosis and treatment policies and performance appraisal standards for public hospitals by quantifying the correlation between process integration and patient experience. It helps drive the transformation of the medical service system toward refinement and patient-centered care (28).

## 7 Prospects

### 7.1 Deepening intelligent construction

In response to the national policies of medical digital transformation and “Internet Plus Healthcare,” the hospital’s current multiple systems (appointment, medical record, finance, etc.) have not achieved interconnection, which affects work efficiency. On the basis of integrating 10 independent systems into 2 platforms, the hospital will build a unified patient service data model relying on the data middle platform to realize full-process data linkage analysis. In the future, it is necessary to deepen the integration of artificial intelligence and digital technologies, such as using natural language processing (NLP) to analyze patient complaints and feedback and identify pain points in real time; introducing machine learning to predict outpatient peaks and dynamically adjust the number of windows; developing intelligent RPA to handle cross-system data entry and release human resources; adopting AI guidance and intelligent medical insurance Q&A to divert consultation pressure, bridge the “digital divide” for older adults patients, and create a “seamless queuing” model. Replacing “patient running around” with “information flow” will further improve service convenience.

### 7.2 Expanding service connotation boundaries

Closely following the medical reform orientation of full-cycle health management, value-added services such as health consultation, chronic disease follow-up, and medication guidance will be added for groups such as older adults and chronic disease patients, extending services from medical treatment processes to full-cycle health management. Psychological counseling specialists will be introduced in response to patients’ emotional pain points to enhance the human-centered nature of services.

### 7.3 Improving data-driven management system

To meet the requirements of refined medical management, a sophisticated management system will be constructed based on the structured data accumulated by the service center. Performance appraisal will be optimized by incorporating “one-time resolution rate” and “patient emotional improvement degree” into assessment indicators linked to compensation, so as to stimulate staff motivation. A closed-loop governance mechanism of “data monitoring–problem positioning–scheme optimization–effect evaluation” will be established to optimize processes through the PDCA cycle. The scope of data collection will be expanded to build prediction models, realizing the transformation of management from “passive response” to “active prevention”.

### 7.4 Promoting standardized model replication

In line with the national policy orientation of homogenizing medical services and promoting high-quality experience, the core content of the four-dimensional integration model of Quanzhou First Hospital will be modularized to form the Operation Guidelines for the Construction of Outpatient One-Stop Services. Differentiated adaptation plans will be provided according to the characteristics of different institutions such as secondary hospitals and community hospitals, with priority given to hospitals with a high proportion of older adults and cross-regional medical insurance patients. This will help solve common industrial pain points and highlight the public welfare attributes of public hospitals.

## 8 Limitations

### 8.1 Limitations in research design

This study adopted a single-center design, and the research subjects were limited to outpatients at Quanzhou First Hospital, which restricts the generalizability of the conclusions to a certain extent. Meanwhile, the research period was relatively short without a long-term follow-up mechanism, making it difficult to observe the long-term effectiveness and potential risk evolution of the four-dimensional integrated service model. In addition, convenience sampling was used for sample recruitment, which may objectively lead to selection bias. The composition of interviewees did not fully cover the characteristics of patient groups with different age groups, geographical distributions and disease types.

### 8.2 Limitations in measurement and data collection

The collection of some key indicators, especially subjective evaluation indicators such as patient satisfaction, mainly relied on the subjective scoring of respondents, which may bring biases in the measurement process. In addition, as an observational study, the research was subject to the Hawthorne effect, meaning that patients and medical staff might change their routine behaviors due to awareness of being observed, thus affecting the objectivity of data. These methodological constraints may exert a certain impact on the accuracy of the research results.

### 8.3 Limitations in causal identification and economic evaluation

This study did not conduct a systematic cost-effectiveness analysis, lacking quantitative assessment of the balance between initial construction investment and long-term benefits of the four-dimensional integration model. Meanwhile, in terms of causal inference, the study could not completely exclude the confounding effects of other parallel reform measures in the hospital during the same period. Therefore, the observed improvement in service quality could not be fully and solely attributed to the implementation of the four-dimensional integrated service model. These factors restrict the accurate judgment of the model’s real effect and economic feasibility.

### 8.4 Directions for future research and improvement paths

Given the above limitations, follow-up research can be further verified and deepened in the following ways: adopting a multi-center collaborative design to enhance the generalizability of conclusions; introducing a control group for comparative analysis when conditions permit; implementing a long-term tracking mechanism to observe the sustainability of the model; and adopting a more rigorous research design framework and economic evaluation methods to conduct a comprehensive and systematic assessment of the model’s effectiveness, promotion feasibility and cost-effectiveness. These improvements will help further consolidate the evidence base of the research and provide more sufficient academic support for the large-scale application and promotion of the model.

## Data Availability

The raw data supporting the conclusions of this article will be made available by the authors, without undue reservation.
